# Mortality Trends in Alzheimer’s Disease in Mississippi, 2011‐2021

**DOI:** 10.1002/alz.095207

**Published:** 2025-01-09

**Authors:** Elizabeth A.K. Jones

**Affiliations:** ^1^ Jackson Heart Study GTEC, Jackson, MS USA

## Abstract

**Background:**

Alzheimer’s disease is the sixth most common cause of death in the United States (U.S.), with one in three adults 65 years of age and older dying of the disease each year. Deaths from Alzheimer’s have more than doubled between 2000 and 2019, killing more adults than both breast cancer and prostate cancer. In 2021, Alzheimer’s disease resulted in 36 deaths per 100,000 in the U.S. In Mississippi, deaths from Alzheimer’s have almost doubled between 2011 and 2021, resulting in 52.9 deaths per 100,000. Women have a higher mortality rate from Alzheimer’s than men. Alzheimer’s is a progressive disease that develops through seven stages. There are effective strategies to prevent the onset of Alzheimer’s.

**Method:**

This paper reviews the risk factors, mortality trends, etiology, and prognosis of Alzheimer’s in Mississippi with a focus on prevention.

**Result:**

The southern diet with foods high in sugar and sodium, along with sedentary and poor lifestyle choices, increases mortality risk from Alzheimer’s disease for women in Mississippi, specifically due to women over 65 having higher rates of obesity and hypertension.

**Conclusion:**

Understanding the epidemiology and risk factors of Alzheimer’s in Mississippi will help inform communities, policies, and programs to prevent disease occurrence.

